# Association between acute phase reactants, interleukin-6, tumor necrosis factor-α, and disease activity in Takayasu’s arteritis patients

**DOI:** 10.1186/s13075-020-02365-y

**Published:** 2020-12-10

**Authors:** Jing Li, Yahong Wang, Yanhong Wang, Ying Wang, Yunjiao Yang, Jiuliang Zhao, Mengtao Li, Xinping Tian, Xiaofeng Zeng

**Affiliations:** 1 Department of Rheumatology, Peking Union Medical College Hospital, Peking Union Medical College and Chinese Academy of Medical Sciences, National Clinical Research Center for Dermatologic and Immunologic Diseases (NCRC-DID), Key Laboratory of Rheumatology and Clinical Immunology, Ministry of Education, Beijing, 100730 China; 2 Department of Ultrasound Imaging, Peking Union Medical College Hospital, Peking Union Medical College and Chinese Academy of Medical Sciences, Beijing, China; 3 Department of Epidemiology and Biostatistics, Institute of Basic Medical Sciences, Chinese Academy of Medical Sciences, School of Basic Medicine, Peking Union Medical College, Beijing, China

**Keywords:** Takayasu arteritis, Angiographic examination, Disease activity, Acute phase reactants, Interleukin-6, Tumor necrosis factor-α

## Abstract

**Background:**

To investigate the association between blood biomarkers and disease activity of Takayasu’s arteritis (TAK) in a follow-up cohort.

**Methods:**

Disease activity was assessed by clinical manifestations and repeated vascular Doppler examinations. The association between erythrocyte sedimentation rate (ESR), serum levels of high-sensitive C-reactive protein (hsCRP), interleukin-6(IL-6), and tumor necrosis factor-α (TNFα) and disease activity were analyzed by logistic regression and survival analysis. Kaplan-Meier method was used to estimate the cumulative remission rate curve, log-rank tests for group comparison, and Cox regression for estimating hazard ratios of these parameters for disease activity.

**Results:**

428 patients were included. 188 patients were in active disease, and 240 patients were in inactive disease at baseline. Elevation of ESR, hsCRP, and IL-6 were associated with active disease at baseline and during follow-up. Cox regression and Kaplan-Meier analysis showed that lower possibility and longer time to remission were associated with elevated ESR (hazard ratio [HR] = 0.32, 80 vs 33 weeks, *p* < 0.001), hsCRP (HR = 0.45, 70 vs 31 weeks, *p* < 0.001), and IL-6 (HR = 0.54, 66 vs 34 weeks, *p* < 0.01) in patients with active disease at baseline, while higher risk and shorter time for relapse were associated with elevated ESR (HR = 2.1, 59 vs 111 weeks, *p* < 0.001), hsCRP (HR = 2.1, 79 vs 113 weeks, *p* < 0.001), IL-6 (HR = 2.5, 64 vs 117 weeks, *p* < 0.001), and TNFα (HR = 2.7, 65 vs 114 weeks, *p* < 0.001) in patients with inactive disease at baseline.

**Conclusions:**

Elevated ESR, CRP, and IL-6 are associated with active disease, lower possibility, and longer time to achieve disease remission. Elevation of any among ESR, CRP, IL-6, and TNFα is associated with high risk and short time for relapse during follow-up.

**Supplementary Information:**

The online version contains supplementary material available at 10.1186/s13075-020-02365-y.

## Background

Takayasu’s arteritis (TAK) is an uncommon systemic vasculitis that involves aorta and its major branches [1]. The assessment of disease activity in TAK patients is very challenging due to its complicated clinical features and lack of reliable biomarkers. Erythrocyte sedimentation rate (ESR) and C-reactive protein (CRP) were widely used acute phase reactants in monitoring disease activity of patients with TAK. However, ESR and CRP are not reliable indicators because the disease could progress in patients with normal ESR and CRP levels [2].

Interleukin-6 (IL-6) and tumor necrosis factor-α (TNFα) have been shown to be involved in the pathogenesis of TAK [3, 4]. Both of them were over-expressed in the diseased aorta and its branches [5]. Therefore, the elevated level of TNFα and IL-6 may be associated with active disease.

Aorta and its major branches are targets of TAK. Wall thickness and mural stenosis are the typical changes in TAK [6]. The progression of or new onset of wall thickness and stenosis are regarded as the gold indicator for active disease [7]. Therefore, if blood biomarkers that are correlated with vascular image progression and clinical active disease manifestations could be found, it would greatly help rheumatologists to make appropriate medical decisions and improve the long-term prognosis of TAK patients. Based on this hypothesis, we conducted this prospective study aimed to explore the association between blood biomarkers with disease activity of TAK, taking vascular changes as the gold standard.

## Patients and methods

### Patient population

Chinese Registry for Systemic Vasculitis (CRSV), based on the CRDC (Chinese Rheumatism Data Center) platform, was developed to collect the clinical information and prognosis data of Chinese patients with systemic vasculitis. The CRSV registry was initiated in July 2013. So far, 118 member centers participated in the registry. Up to April 30, 2019, 1150 patients with TAK were registered in CRSV. All the registered patients must fulfill the 1990 American College of Rheumatology (ACR) classification criteria for Takayasu’s arteritis [8]. Among them, 588 patients were registered and followed-up in Peking Union Medical College Hospital (PUMCH). At the time of registration, computed tomography angiography (CTA) was performed in all patients to confirm the diagnosis and evaluate the extent of artery involvement.

Patients in this study were followed every 1 and 3 to 6 months according to patients’ disease activity status. At each visit, ESR, blood hs-CRP, TNFα, and IL-6 levels were tested. Disease activity was comprehensively assessed based on clinical presentations and Doppler examination results at each visit. Repeated vascular Doppler examinations were performed every 3 to 6 months according to the patient’s disease activity status. Patients with active disease in the first visit were followed-up 1–3 months later. Patients were followed every 6 months if they were assessed to be in inactive disease at the first visit. All patients had vascular Doppler imaging examination at baseline and every follow-up visit.

This study was approved by the Institutional Review Board of Peking Union Medical College Hospital (S-478), Beijing, China. Written informed consent was obtained from all participants, and the study was performed in accordance with the Declaration of Helsinki. Personal information was protected and kept anonymous in CRSV database.

### Clinical data

The demographic data and medical histories were collected when patients were registered in CRSV. The clinical manifestations, Birmingham Vasculitis Activity Score (BVAS), Vasculitis Damage Index (VDI) score, laboratory tests (including ESR, serum levels of hsCRP, IL-6, and TNFα), imaging findings (including serial vascular Doppler imaging examination, CTA, etc.), and treatment regimen were recorded at each visit. Outcomes, complications, and adverse events related to medications were recorded if occurred.

Serum levels of IL-6 and TNF-alpha were detected with Siemens LK6P1 and LKNF1 assay kit by IMMULITE/IMMULITE 1000 system at each visit in our center.

Repeated vascular Doppler of carotid arteries, vertebral arteries, subclavian arteries, axillary artery, ulnar and radial artery, abdominal aorta, renal arteries, celiac arteries, and mesangial arteries were performed and compared with former results to identify changes. Since the reconstruction of the vessel wall is a continued process, the changes of vascular images might be found later than that of clinical disease flare. The information of image results was stored in the electronic clinical information system of our center.

### Evaluation of disease activity

Disease activity of patient with TAK was evaluated by 2 senior rheumatologists who have more than 5 years of experiences in taking care of TAK patients. They were responsible for judging the disease activity based on comprehensive assessment of clinical manifestations and serial vascular Doppler imaging findings at each visit. In this study, all vascular examinations were performed by 2 vascular Doppler specialists who had ever been trained for large vessel Doppler examination. They were blind to the lab test results of patients. The “image active disease” was defined as new occurrence of stenosis or occlusion or dilatation (including aneurysm), worsen mural thickness or stenosis. The definition of active disease was also defined as the presence of the following symptoms or signs after other causes were excluded (“clinical active disease”): fever, weight loss, fatigue and/or arthralgia/arthritis/myalgia, new onset or aggravated symptoms of ischemia (including limb claudication, stroke, dizziness, syncope, severe abdominal pain, myocardial infarction, or angina), acute visual symptoms such as amaurosis fugax or diplopia, new onset hypertension, new onset of vessel bruit, new loss of pulses, carotidynia or tenderness of vessels, and other conditions judged by the 2 senior rheumatologists that increase the dosage of corticosteroid and/or necessary addition of immunosuppressive drugs. Patients with any among “clinical active disease”, “image active disease”, or judged by the rheumatologist as active were defined as “active disease.” If the patient had none of the clinical manifestations for active disease and did not have active image presentations, or judged by the rheumatologist as in inactive disease, then the patient was defined as “inactive disease.” Flare was defined when the patient’s disease status changed from “inactive disease” to “active disease,” and remission was defined when the patient’s disease status changed from “active disease” to “inactive disease.”

### Visits during follow-up

The visit when all the above blood parameters were tested and data were collected from serial vascular Doppler imaging examination for future comparison was defined as the baseline visit. Then, patients were followed-up at 1- and 3 to 6-month intervals depending on the patient’s situation and disease activity status was evaluated by the 2 rheumatologists. At each follow-up visit, comprehensive history taking and physical examination were done, all the parameters were tested, and vascular Doppler imaging examination was repeated every 3 to 6 months.

### Statistical analysis

Continuous variables were expressed as the mean ± standard deviation for the data in a normal distribution and median (quantile 1, quantile 3) for the non-normal distributed data. Kolmogorov-Smirnov test was used to test data normality. If normal distribution was satisfied, *t* test was used to compare between groups. Categorical variables were expressed as absolute numbers and percentages, and chi-squared test was used to compare between groups.

In our analysis, serum level of TNFα was categorized into normal and abnormal groups by the upper limit of normal range in healthy population (≤ 8.1 pg/ml), so was serum level of IL-6 (upper limit of normal range ≤ 5.9 pg/ml), hsCRP (upper limit of normal range ≤ 8 mg/L), and ESR (upper limit of normal range ≤ 20 mm/1st hour).

The univariate logistic regression analysis was performed to investigate the association between these parameters (ESR, hsCRP, IL-6, and TNFα) and disease activity at baseline. Furthermore, sensitivity, specificity, and the 95% confidence intervals were calculated. For follow-up data, univariate Cox regression model was used to explore the association of ESR, hsCRP, IL-6, and TNFα with disease activity in the follow-up visits. Positive predictive values and negative predictive values, as well as the 95% confidence intervals were also represented. Kaplan-Meier method was conducted to describe the curve of cumulative remission rate, and log-rank tests were used to compare cumulative remission curves.

For patients with active disease at baseline but turned inactive during follow-up, the follow-up time in survival analysis was the duration from the date of baseline visit to the date of visit when inactive disease was first achieved. For those patients who were still in active state in later follow-up visits, their follow-up time was defined from the date of baseline visit to the end of the study.

For patients who were in inactive disease at baseline but changed to active diseases during follow-up, their follow-up duration was defined from the date of baseline visit to the date of visit for first relapse. For patients who remained in inactive during follow-up, their follow-up duration was defined from the date of baseline visit to the end of the study.

A two-sided *p* value less than 0.05 was considered to be statistically significant, and the odds ratio (OR) or hazard ratio (HR) with a 95% confidence interval (CI) were also calculated. Analysis was performed with the SAS software (version 9.0, SAS Institute, Cary, NC, USA).

## Results

### Demographic data, clinical features, and laboratory findings of patients

**Fig. 1:**
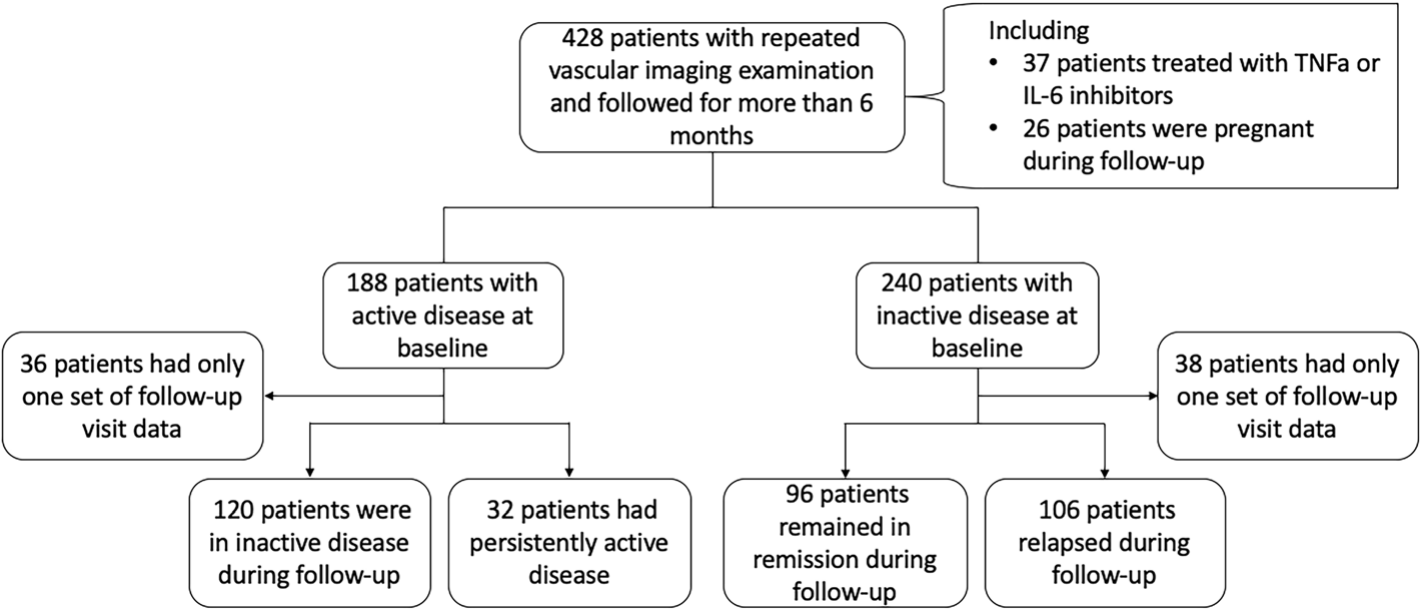
The flow chart of the study and data collection. The patients with Takayasu’s arteritis in this study were all from the Chinese Registry of Systemic Vasculitis (CRSV). Clinical data of 428 patients with repeated vascular imaging examination and followed more than 6 months were collected

428 patients were included in this study prospectively from PUMCH. Based on the definition described above, 188 patients were defined as with “active disease” and 240 patients were defined as with “inactive disease” at baseline (Fig. 1).

Demographic data, laboratory test results, and repeated Doppler image results of these patients at baseline were summarized in Table 1. There was no significant difference between these two groups in age (*p* = 0.26), gender (*p* = 0.63), and disease durations (*p* = 0.51). In active group at baseline, the median follow-up time (33 months) was shorter than in inactive group (64 months) (*p* < 0.001) at baseline (Table 1).

**Table 1:** The demographic data, BVAS and VDI scores, laboratory test results, and Doppler image results of 428 patients with Takayasu's arteritis at baseline

	Total (*n* = 428)	Active group (*n* = 188)	Inactive group (*n* = 240)	*p* value
Age, years	31.8 ± 9.5	31.8 ± 9.9	31.8 ± 9.1	0.26
Female, *n* (%)	393 (91.8)	174 (92.6)	219 (91.3)	0.63
Disease duration, months (Q1, Q3)	53 (29, 99)	52 (29, 107)	54 (31, 96)	0.51
Median follow-up time, months (Q1, Q3)^#^	49 (28, 85)	33 (25, 61)	64 (34, 98)	< 0.001
BVAS scores	6.7 ± 3.7	7.0 ± 3.7	6.4 ± 3.7	0.10
VDI scores	2.7 ± 1.8	2.8 ± 2.0	2.6 ± 1.6	0.28
*Laboratory test results*				
ESR, mm/1st hour	17.1 ± 19.6	25.6 ± 25.1	10.4 ± 9.4	< 0.001
ESR > 20 mm/1st hour, *n* (%)	106/421* (25.2)	78/186* (41.9)	28/235* (11.9)	< 0.001
hsCRP, mg/L	13.2 ± 26.7	25.6 ± 36.2	3.4 ± 5.5	< 0.001
hsCRP > 8 mg/L, *n* (%)	141/410* (34.4)	115/181* (63.5)	26/229* (11.4)	< 0.001
IL-6, pg/ml	13.9 ± 40.0	21.2 ± 42.3	7.4 ± 36.7	0.003
IL-6 > 5.9 pg/ml, *n* (%)	82/241* (34.0)	64/114* (56.1)	18/127* (14.2)	< 0.001
TNFα, pg/ml	16.3 ± 34.2	19.9 ± 44.3	13.0 ± 21.3	0.02
TNFα > 8.1 pg/ml, *n* (%)	104/238* (43.7)	54/112* (48.2)	50/126* (39.7)	0.19
*Repeated vascular Doppler image results compared to former results*				
Stable	175 (40.1%)	27 (14.4%)	148 (61.7%)	
Improved	15 (3.5%)	2 (1.1%)	13 (5.4%)	
Progressed	238 (55.6%)	159 (84.6%)	79 (32.9%)	

Abbreviation: *ESR* erythrocyte sedimentation rate, *hsCRP* high sensitive C-reactive protein, *IL-6* interleukin-6, *TNFα* tumor necrosis factor-α

^#^Among the follow-up patients

*Actually detected

Comparisons between active and inactive groups at baseline showed significant differences in the levels of ESR, hsCRP, IL-6, and TNFα. While comparing the proportions of patients with elevated level of these four parameters, no significant difference was found between the two groups for TNFα (*p* = 0.19) only, while there was a significant difference in the other three parameters (Table 1).

There was no significant difference in clinical manifestations between these two groups, as well as in BVAS scores and the VDI scores. The results of comparison between these two groups were summarized in Supplementary Table-S1.

### Association between laboratory findings with disease activity at baseline

Elevated level of ESR (OR = 5.34, 95%CI 3.27 ~ 8.72), hsCRP (OR = 13.60, 95%CI 8.18 ~ 22.62), and IL-6 (OR = 7.75, 95%CI 4.17 ~ 14.42) of 428 patients were found to be associated with active disease activity at baseline in univariate logistic regression. But elevated level of TNFα (OR = 1.42, 95%CI 0.85 ~ 2.37) was not associated with active disease at baseline (Table 2). After excluding patients treated with TNFα inhibitors or IL-6 inhibitor and patients in pregnancy, the results remained the same (Supplementary Table-S2).

**Table 2:** Association between ESR, hsCRP, IL-6 and TNFα with disease activity at baseline in logistic regression. Results of univariate logistic regression analysis were shown in this table, which analyzed the association between ESR, hsCRP, IL-6 and TNFα with disease activity at baseline in 428 patients

	Odds ratio (95% CI)	*p* value	Sensitivity (95% CI)	Specificity (95% CI)
ESR (> 20 mm/1st hour)	5.34 (3.27 ~ 8.72)	< 0.001	41.9% (34.8%–49.4%)	88.1% (83.2%–91.9%)
hsCRP (> 8 mg/L)	13.60 (8.18 ~ 22.62)	< 0.001	63.5% (56.1%–70.6%)	88.7% (83.8%–92.5%)
IL-6 (> 5.9 pg/ml)	7.75 (4.17 ~ 14.42)	< 0.001	56.1% (46.5%–65.4%)	85.8% (78.5%–91.4%)
TNFα > 8.1 pg/ml)	1.42 (0.85 ~ 2.37)	0.19	48.2% (38.7%–57.9%)	60.3% (51.2%–68.9%)

Abbreviation: *ESR* erythrocyte sedimentation rate, *hsCRP* high sensitive C-reactive protein, *IL-6* interleukin-6, *TNFα* tumor necrosis factor-α, *CI* confidence interval

Furthermore, sensitivity and specificity were also be reported in Table 2. The specificity was 88.1% for ESR, 88.7% for hsCRP, 85.8% for IL-6, and 60.3% for TNFα. And the sensitivity was 41.9% for ESR, 63.5% for hsCRP, 56.1% for IL-6, and 48.2% for TNFα.

### Survival analysis of ESR, hsCRP, IL-6, and TNFα with disease activity during follow-up

In this study, 152 patients of the active group and 202 patients of the inactive group at baseline were followed-up and included in the survival analysis (Fig. 1). Among 152 patients of the active group, 120 patients (79.0%) achieved inactive disease in the follow-up visit, and the median time to achieve inactive disease was 35 weeks (95% CI 31–40 weeks). Moreover, in 202 patients of the inactive group, 106 patients (52.5%) were found to relapse in the follow-up visit. The median time to relapse was 94 weeks (95%CI 75−109 weeks).

**Fig. 2:**
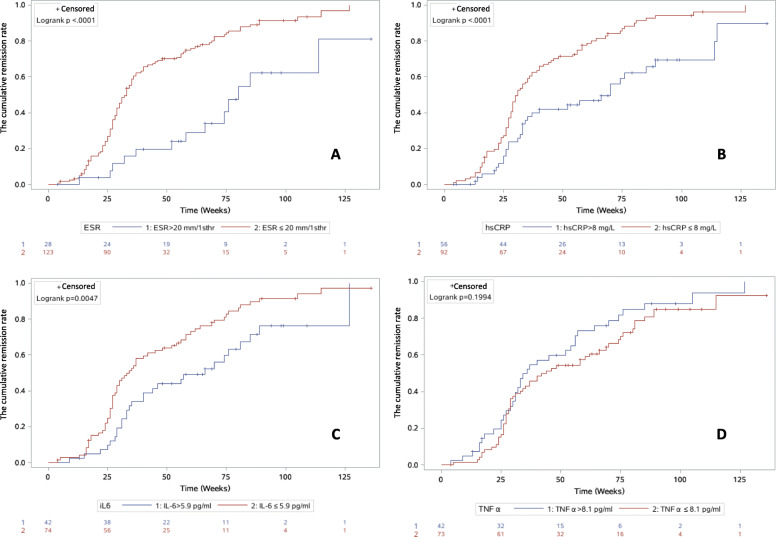
The cumulative remission rate curves by using Kaplan-Meier analysis for the active group at baseline. This figure demonstrated the cumulative remission rate curves by using Kaplan-Meier analysis of 152 patients in active group at baseline, with further follow-up data. Elevated levels of ESR (2A, 80 vs 33 weeks, *p* < 0.0001), hsCRP (2B, 70 vs 31 weeks, *p* < 0.0001), and IL-6 (2C, 66 vs 34 weeks, *p* < 0.01) at baseline were associated with longer time to achieve remission in those patients with active disease at baseline

**Fig. 3:**
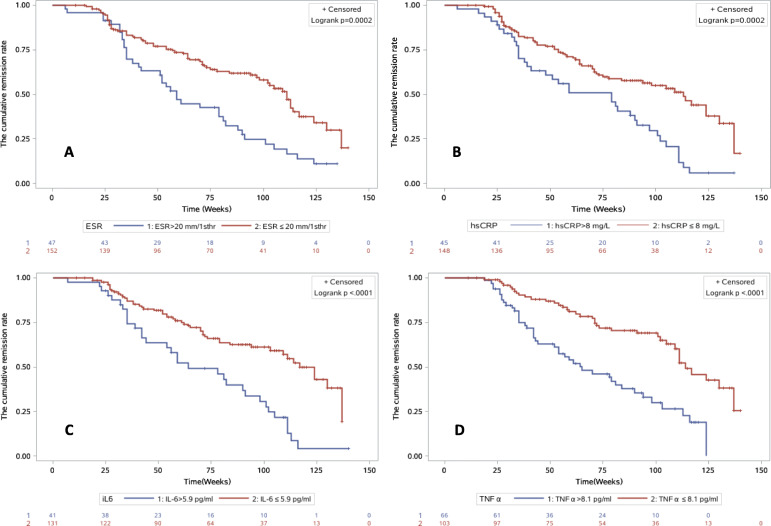
The cumulative remission rate curves by using Kaplan-Meier analysis for the inactive group at baseline. This figure demonstrated the cumulative remission rate curves by using Kaplan-Meier analysis of 202 patients in inactive group at baseline with further follow-up data. Elevated levels of ESR (3A, 59 vs 111 weeks, *p* < 0.001), hsCRP (3B, 79 vs 113 weeks, *p* < 0.001), IL-6 (3C, 64 vs 117 weeks, *p* < 0.0001), and TNFα (3D, 65 vs 114 weeks, *p* < 0.0001) were associated with shorter time to relapse in those patients with inactive disease at baseline

As showed in Fig. 2, elevated levels of ESR (2A, 80 vs 33 weeks), hsCRP (2B, 70 vs 31 weeks), and IL-6 (2C, 66 vs 34 weeks) at baseline were associated with longer time to achieve remission in those patients with active disease at baseline (*p* < 0.01) (Fig. 2, Supplementary Table-S3). Elevated levels of ESR (3A, 59 vs 111 weeks), hsCRP (3B, 79 vs 113 weeks), IL-6 (3C, 64 vs 117 weeks), and TNFα (3D, 65 vs 114 weeks) were associated with shorter time to relapse in those patients with inactive disease at baseline (*p* < 0.001). (Fig. 3, Supplementary Table-S4).

Cox regression also revealed that elevated levels of ESR (HR: 0.32, 95%CI 0.18–0.58), hsCRP (HR: 0.45, 95%CI 0.30–0.68), and IL-6 (HR: 0.54, 95%CI 0.34–0.84) were associated with lower possibility to achieve an inactive state in patients with active group at baseline. In addition, the elevated level of ESR (HR: 2.11, 95%CI 1.41–3.16), hs-CRP (HR: 2.13, 95%CI 1.42–3.20), IL-6 (HR: 2.50, 95%CI 1.60–3.91), and TNFα (HR: 2.65, 95%CI 1.69–4.15) were found to be associated with higher risk for disease relapse in patients of the inactive group at baseline (Table 3).

**Table 3:** Cox regression analysis on the association of laboratory results with disease activity in follow-up visits. Results of Cox regression analysis were shown in this table, which analyzed the association of ESR, hsCRP, IL-6, and TNFα with inactive disease or relapse in follow-up visits among 354 Takayasu’s arteritis patients who were followed-up

		Disease activity in follow-up visits		***p*** value	HR (95% CI)	Positive predictive value	Negative predictive value
		Inactive	Active			(95% CI)	(95% CI)
**152 patients in active group at baseline**							
Number of patients		120	32				
ESR	(≤ 20 mm/1st hour)	106 (86.2%)	17 (13.8%)	< 0.001	1.00	53.6% (33.9–72.5%)	86.2% (78.8–91.7%)
	(> 20 mm/1st hour)	13 (46.4%)	15 (53.6%)		0.32 (0.18–0.58)		
hsCRP	(≤8 mg/L)	84 (91.3%)	8 (8.7%)	< 0.001	1.00	42.7% (29.7-56. 8%)	91.3% (83.6–96.2%)
	(> 8 mg/L)	32 (57.1%)	24 (42.9%)		0.45 (0.30–0.68)		
IL-6	(≤ 5.9 pg/ml)	65 (87.8%)	9 (12.2%)	0.006	1.00	33.3% (19.6–49.6%)	87.8% (78.2–94.3%)
	(> 5.9 pg/ml)	28 (66.7%)	14 (33.3%)		0.54 (0.34–0.84)		
TNFα	(≤ 8.1 pg/ml)	56 (76.7%)	17 (23.3%)	0.25	1.00	14.3% (5.4–28.5%)	76.7% (65.4–85.8%)
	(> 8.1 pg/ml)	36 (85.7%)	6 (14.3%)		1.31 (0.86–2.00)		
**202 patients in inactive group at baseline**							
Number of patients		96	106				
ESR	(≤ 20 mm/1^st^hr)	87 (57.3%)	65 (42.8%)	< 0.001	1.00	80.9% (66.7–90.9%)	57.2% (49.0–65.2%)
	(> 20 mm/1^st^hr)	9 (19.2%)	38 (80.9%)		2.11 (1.41–3.16)		
hsCRP	(≤ 8 mg/L)	84 (56.8%)	64 (43.3%)	< 0.001	1.00	82.2% (68.0–92.0%)	56.8% (48.4–64.9%)
	(> 8 mg/L)	8 (17.8%)	37 (82.2%)		2.13 (1.42–3.20)		
IL-6	(≤5.9 pg/ml)	80 (61.1%)	51 (38.9%)	< 0.001	1.00	78.1% (62.4–89.4%)	61.1% (52.2–69.5%)
	(> 5.9 pg/ml)	9 (22.0%)	32 (78.1%)		2.50 (1.60–3.91)		
TNFα	(≤8.1 pg/ml)	65 (63.1%)	38 (36.9%)	< 0.001	1.00	65.2% (52.4–76.5%)	63.1% (53.0–72.4%)
	(> 8.1 pg/ml)	23 (34.9%)	43 (65.2%)		2.65 (1.69–4.15)		

Abbreviation: *HR* hazard ratio, *ESR* erythrocyte sedimentation rate, *hsCRP* high sensitive C-reactive protein, *IL-6* interleukin-6, *TNFα* tumor necrosis factor-α, *CI* confidence interval

The positive predictive value and negative predictive value are presented in Table 3. In active patients at baseline, compared to the positive predictive values, higher negative predictive values were found to be 86.2% for ESR, 91.3% for hsCRP, 87.8% for IL-6, and 76.7% for TNF-α. On the contrary, in inactive patients at baseline, higher positive predictive value was found to be 80.9% for ESR, 82.2% for hsCRP, 78.1% for IL-6, and 65.2% for TNF-α.

Repeated vascular Doppler image results of patients at follow-up visits are shown in Table 4.

**Table 4:** Repeated vascular Doppler imaging results of 354 patients with Takayasu’s arteritis in follow-up visits

	Stable	Improved	Progressed
**152 patients in active group at baseline**			
120 patients achieved inactive disease	72 (60.0%)	15 (12.5%)	33 (27.5%)
32 patients sustained active disease	9 (28.1%)	5 (15.6%)	18 (56.3%)
**202 patients in inactive group at baseline**			
96 patients remained in remission	53 (55.2%)	30 (31.3%)	13 (13.5%)
106 patients relapsed	17 (16.0%)	3 (2.8%)	86 (81.1%)

## Discussion

TAK is characterized by relapse and remission in the course of the disease. The goal of the management of TAK is to induce the disease into remission and maintain the disease in persistent inactive condition in order to preserve blood supply and improve the long-term outcome. However, assessing disease activity is difficult and very challenging. Although a number of assessment instruments or biomarkers had been developed to assess disease activity, none of them were reliable or easy to apply in clinical practice. Birmingham Vasculitis Activity Score (BVAS) [9], physician global assessment (PGA) [10], Disease Extent Index-Takayasu (DEI.Tak) [11], and Indian Takayasu Activity Score (ITAS) were the most commonly used instruments [12]. BVAS is not well suitable for disease activity assessment for large vessel vasculitis, so it is not routinely used for disease activity evaluation for TAK in clinical practice. The ITAS was derived from the DEI.Tak. These two instruments only include clinical symptoms and signs but do not include imaging findings and acute phase reactants [11, 12]. Though the ITAS had shown good correlation with PGA in an Indian center [12], it is time-consuming and not easy to apply in daily clinical practice.

ESR and CRP are the most commonly used biomarkers for the assessment of disease activity of TAK. The sensitivity and specificity of ESR for active TAK are 72% and 56%, respectively [13]. Although CRP has a sensitivity of 71.4% and specificity of 100% for active disease in a study [14], CRP may elevate nonspecifically in tissue inflammation and infection, so it may not accurately reflect the disease status. High sensitive CRP (hs-CRP) is developed to detect CRP in a more sensitive way, but had the same limitations as CRP. Furthermore, TAK could relapse or progress in the absence of elevated CRP and ESR. Many biomarkers had been investigated to assess disease activity of TAK in addition to ESR and CRP. Levels of serum IL-6 and TNFα were found to be elevated in patients with TAK, and IL-6 levels were higher in those with active disease [15]. However, so far, no study has been reported to investigate the association between these parameters with disease activity during follow-up study or to take vascular image changes as the major evidence into consideration for disease activity evaluation. In this study, we explored the role of ESR, hsCRP, IL-6, and TNFα in the assessment of disease activity in a large follow-up cohort of Chinese TAK patients.

In our study, patients were followed-up at 3- to 6-month interval regularly. At each visit, symptoms and signs were recorded and examined, vascular Doppler imaging examinations were performed and levels of acute phase reactants, and TNFα and IL-6 were tested. All these enabled us to evaluate patient’s disease activity more comprehensively and accurately than basing our evaluation solely on the acute phase reactants levels.

In this study, the inflammatory parameters, including the elevation of ESR, hsCRP, and IL-6, were found to be associated with active disease of TAK by logistic regression. This result was consistent with the study by Tamura and co-researchers [15]. Furthermore, in this study, we found that ESR, hsCRP, TNFα, and IL-6 were associated with active disease during follow-up by survival analysis, both in Cox regression and Kaplan-Meier models. This suggested that test for these 4 parameters might provide evidence for rheumatologists to determine disease activity of TAK patients.

Our study also showed that elevated ESR, hs-CRP, IL-6, and TNFα were independently associated with disease relapse during follow-up. In cohort study of Comarmond and co-researchers, elevated level of ESR and CRP was associated with the relapse during follow-up [16]. Our study not only confirmed their results in a much larger patient population, but also showed that ESR, hsCRP, TNFα, and IL-6 were associated with a higher risk for relapse during follow-up when compared with patients with normal levels of ESR, hsCRP, TNFα, and IL-6. This is very helpful in patients whose seral ESR and/or hs-CRP level were not elevated. Our results also demonstrated that patients who were currently inactive, with elevated ESR, CRP, IL-6, and TNFα would relapse in a shorter time during follow-up compared to patients with normal ESR, hs-CRP, IL-6, and TNFα. Based on this result, we might conclude that these parameters not only provided clues for active disease, but also predicted upcoming relapse in near future.

The elevation of IL-6 was not only associated with active disease of TAK at baseline, but also associated with active disease during follow-up, including longer time to achieve remission in patients of active group at baseline and shorter time to relapse in inactive group at baseline. While elevation of TNFα was found to be associated with shorter time to relapse in patients with inactive disease at baseline, it is not associated with longer time to achieve inactive state. Previous studies had shown that these two indices are elevated in active TAK patients [15] as compared to healthy controls or patients in remission [17], but did not investigate their association with relapse during follow-up. Results of this study suggested that detection of TNFα and IL-6 was not only useful to help evaluate disease activity in the present visit, but also helpful to identify patients at high risk of relapse.

There are some limitations in our study. First, the treatment was not considered as a control variable in the models. For patients with inactive disease at baseline, the treatment would be kept the same or adjusted in order to get persistent remission even though the dosage of glucocorticosteroids was tapered to minimize the side effects of the drug. Therefore, the effect of drugs was minimized in this group of patients. However, in patients with active disease at baseline, the dosage of glucocorticosteroid would be increased or the DMARDs were added to induce remission. The adjustment of therapy was individualized based on treatment history and disease severity, which was difficult to standardize. Therefore, the impact of drugs could not be put into our study as an explanatory variable, due to the categories of medication, varied dosages of drugs, and limited sample size. Taking the treatment changes during follow-up visits into consideration, we classified the patients into active and inactive disease at baseline in order to reduce the confounding of treatment to some extents. Doppler ultrasound cannot visualize the thoracic aorta, so it has some limitations in detecting vascular changes in the ascending and thoracic aorta. However, it is more accessible, much cheaper than CTA or MRA, and easier to be performed repeatedly. For those patients with thoracic aorta involvement, we repeated CTA every year or 2 years if there is no evidence of active disease or recurrence, or repeated CTA with shorter interval if there are symptoms and signs suggestive of active disease in this part of aorta (such as persistent back pain or pain at swallow with no other identifiable causes). The concern is that CTA would cause radiological harm particularly for young women with the desire of giving birth and may compromise renal function. Therefore, we would repeat CTA when there was clinical symptoms, signs suggesting thoracic aorta involvement, or when the patient needs to have pre-surgery evaluation.

## Conclusions

Elevated level of ESR, CRP, and IL-6 are associated with active disease of TAK in this study. During follow-up, elevated levels of any of these three indices are associated with longer time and lower possibility to achieve inactive disease in patients with active disease in the present visit. Furthermore, elevated levels of ESR, CRP, IL-6, and TNFα are found to be associated with a higher risk for relapse and shorter time to relapse in patients with currently inactive disease. The results of this study suggest that ESR, CRP, IL-6, and TNFα should be tested and monitored for disease activity evaluation during the follow-up of TAK patients. Testing serum TNFα and IL-6 is particularly helpful in patients with normal acute phase reactants.

## Supplementary Information

**Additional file 1: Supplementary Table S1.** The clinical manifestation of 428 patients with Takayasu’s arteritis at baseline.

**Additional file 2: Supplementary Table S2.** 1 Association between ESR, hsCRP, IL-6 and TNFα with disease activity at baseline in univariate logistic regression, excluded patients who were treated with TNFα inhibitors or IL-6 inhibitor and/or patients in pregnancy. **Supplementary Table S2.2.** Association between ESR, hsCRP, IL-6 and TNFα with disease activity at baseline in univariate logistic regression, excluded patients who were treated with TNFα inhibitors or IL-6 inhibitor.

**Additional file 3: Supplementary Table S3.** The results of survival analysis by using Kaplan-Meier analysis in 152 patients with Takayasu’s arteritis in active group (Fig. 2A-D) at baseline with further follow-up data.

**Additional file 4: Supplementary Table S4.** The results of survival analysis by using Kaplan-Meier analysis in 202 patients with Takayasu’s arteritis in inactive group (Fig. 3A-D) at baseline with further follow-up data.

## Data Availability

The datasets used and/or analyzed during the current study are available from the corresponding author on reasonable request.

## Abbreviations

TAK: Takayasu’s arteritis
ESR: Erythrocyte sediment rate
CRP: C-reactive protein
hs-CRP: High-sensitive C-reactive protein
IL-6: Interleukin-6
TNFα: Tumor necrosis factor-α
HR: Hazard ratio
CRSV: Chinese Registry for Systemic Vasculitis
CRDC: Chinese Rheumatism Data Center
ACR: American College of Rheumatology
PUMCH: Peking Union Medical College Hospital
BVAS: Birmingham Vasculitis Activity Score
VDI: Vasculitis Damage Index
CTA: Computed tomography angiography
OR: Odds ratio
CI: Confidence interval
PGA: Physician global assessment
DEI.Tak: Disease Extent Index-Takayasu
ITAS: Indian Takayasu Activity Score
