# Supplementary material for: Association between acute phase reactants, interleukin-6, tumor necrosis factor-α, and disease activity in Takayasu’s arteritis patients
Source: Arthritis Res Ther. 2020 Dec 10;22:285. doi: 10.1186/s13075-020-02365-y (PMC7726865; doi:10.1186/s13075-020-02365-y)
Supplement: Supplementary file 1 — Additional file 1: Supplementary Table S1. The clinical manifestation of 428 patients with Takayasu’s arteritis at baseline. [file 13075_2020_2365_MOESM1_ESM.doc]

**Supplementary Table -S1 The clinical manifestation of 428 patients with Takayasu’s arteritis at baseline.**

|  | **Total**  **(n=428)** | **Active group**  **(n=188)** | **Inactive group**  **(n=240)** | ***P*-value** |
| --- | --- | --- | --- | --- |
| Age, years | 31.8 ± 9.5 | 31.8 ± 9.9 | 31.8 ± 9.1 | 0.26 |
| BVAS scores | 6.7 ± 3.7 | 7.0 ± 3.7 | 6.4 ± 3.7 | 0.10 |
| VDI scores | 2.7 ± 1.8 | 2.8 ± 2.0 | 2.6 ± 1.6 | 0.28 |
| Fever | 4 (0.9%) | 3 (1.6%) | 1 (0.4%) | 0.32 |
| Weight loss | 1 (0.2%) | 1 (0.5%) | 0 | 1.00 |
| Arthralgia | 1 (0.2%) | 0 | 1 (0.4%) | 1.00 |
| Skin rash | 2 (0.5%) | 0 | 2 (0.8%) | 0.51 |
| Headache | 3 (0.7%) | 2 (1.1%) | 1 (0.4%) | 0.58 |
| Carotidynia | 15 (3.5%) | 7 (3.7%) | 8 (3.3%) | 1.00 |
| Claudication | 62 (14.5%) | 30 (16.0%) | 32 (13.3%) | 0.49 |
| Visual loss | 14 (3.3%) | 9 (4.8%) | 5 (2.1%) | 0.17 |
| Ischemic stroke | 29 (6.8%) | 15 (8.0%) | 14 (5.8%) | 0.44 |
| Decreased pulsation | 213 (49.8%) | 95 (50.5%) | 118 (49.2%) | 0.85 |
| Asymmetric blood pressure | 226 (52.8%) | 101 (53.7%) | 125 (52.1%) | 0.77 |
| Hypertension | 95 (22.2%) | 35 (18.6%) | 60 (25.0%) | 0.13 |
| Bruit of carotid arteries | 283 (66.1%) | 133 (70.7%) | 150 (62.5%) | 0.08 |
| Abdominal vessel bruits | 34 (7.9%) | 13 (6.9%) | 21 (8.8%) | 0.59 |
| Aortic insufficiency | 111 (25.9%) | 52 (27.7%) | 59 (24.6%) | 0.51 |
| Vessel dilation (aneurysm) | 73 (17.1%) | 35 (18.6%) | 38 (15.8%) | 0.52 |
| Stenosis of vessels | 405 (94.6%) | 178 (94.7%) | 227 (94.6%) | 1.00 |
| Angioplasty operations | 120 (28.0%) | 51 (27.1%) | 69 (28.8%) | 0.75 |
