# Supplementary material for: Association between acute phase reactants, interleukin-6, tumor necrosis factor-α, and disease activity in Takayasu’s arteritis patients
Source: Arthritis Res Ther. 2020 Dec 10;22:285. doi: 10.1186/s13075-020-02365-y (PMC7726865; doi:10.1186/s13075-020-02365-y)
Supplement: Supplementary file 2 — Additional file 2: Supplementary Table S2. 1 Association between ESR, hsCRP, IL-6 and TNFα with disease activity at baseline in univariate logistic regression, excluded patients who were treated with TNFα inhibitors or IL-6 inhibitor and/or patients in pregnancy. Supplementary Table S2.2. Association between ESR, hsCRP, IL-6 and TNFα with disease activity at baseline in univariate logistic regression, excluded patients who were treated with TNFα inhibitors or IL-6 inhibitor. [file 13075_2020_2365_MOESM2_ESM.docx]

**Supplementary table – S2.1 Association between ESR, hsCRP, IL-6 and TNFα with disease activity at baseline in univariate logistic regression, excluded patients who were treated with TNFα inhibitors or IL-6 inhibitor and/or patients in pregnancy.**

|  | **Odds Ratio** | **95% Confidence interval** | **p-value** |
| --- | --- | --- | --- |
| **Exclude patients with medication of TNFα or IL6 inhibitor and/or pregnancy (N=382)** | | | |
| ESR (>20 mm/1^st^hr) | 5.56 | 3.23 ~ 9.54 | <0.001 |
| hsCRP (>8 mg/L) | 15.33 | 8.75 ~ 26.89 | <0.001 |
| IL-6 (>5.9 pg/ml) | 7.96 | 4.08 ~ 15.51 | <0.001 |
| TNFα (>8.1 pg/ml) | 1.42 | 0.83 ~ 2.44 | 0.21 |

**Supplementary table – S2.2 Association between ESR, hsCRP, IL-6 and TNFα with disease activity at baseline in univariate logistic regression, excluded patients who were treated with TNFα inhibitors or IL-6 inhibitor.**

|  | **Odds Ratio** | **95% Confidence interval** | **p-value** |
| --- | --- | --- | --- |
| **Exclude patients with medication of TNFα or IL6 inhibitor (N=394)** | | | |
| ESR (>20 mm/1^st^hr) | 5.23 | 3.10 ~ 8.83 | <0.001 |
| hsCRP (>8 mg/L) | 15.43 | 8.90 ~ 26.75 | <0.001 |
| IL-6 (>5.9 pg/ml) | 8.32 | 4.29 ~ 16.16 | <0.001 |
| TNFα (>8.1 pg/ml) | 1.42 | 0.83 ~ 2.42 | 0.20 |
