# Supplementary material for: Association between acute phase reactants, interleukin-6, tumor necrosis factor-α, and disease activity in Takayasu’s arteritis patients
Source: Arthritis Res Ther. 2020 Dec 10;22:285. doi: 10.1186/s13075-020-02365-y (PMC7726865; doi:10.1186/s13075-020-02365-y)
Supplement: Supplementary file 3 — Additional file 3: Supplementary Table S3. The results of survival analysis by using Kaplan-Meier analysis in 152 patients with Takayasu’s arteritis in active group (Fig. 2A-D) at baseline with further follow-up data. [file 13075_2020_2365_MOESM3_ESM.docx]

**Supplementary table – S3 The results of survival analysis by using Kaplan-Meier analysis in** **152 patients with Takayasu’s arteritis in active group (Figure 2A-D) at baseline with further follow-up data.**

| **Patients numbers (N=152)** | | | | **Median duration to achieve remission (weeks)** | **95%Confidence interval (weeks)** | **P-value** |
| --- | --- | --- | --- | --- | --- | --- |
| 2A | ESR | (≤20 mm/1^st^hr) | 33 | | 29-35 | <0.001 |
|  |  | (>20 mm/1^st^hr) | 80 | | 58-* |  |
| 2B | hsCRP | (≤8 mg/L) | 31 | | 29-35 | <0.001 |
|  |  | (>8 mg/L) | 70 | | 34-85 |  |
| 2C | IL-6 | (≤5.9 pg/ml) | 34 | | 28-42 | 0.005 |
|  |  | (>5.9 pg/ml) | 66 | | 35-81 |  |
| 2D | TNFα | (≤8.1 pg/ml) | 43 | | 31-66 | 0.27 |
|  |  | (>8.1 pg/ml) | 36 | | 30-54 |  |

***** The estimation was restricted because of limited sample size of 28 and censored 15 of them.
