# Supplementary material for: Association between acute phase reactants, interleukin-6, tumor necrosis factor-α, and disease activity in Takayasu’s arteritis patients
Source: Arthritis Res Ther. 2020 Dec 10;22:285. doi: 10.1186/s13075-020-02365-y (PMC7726865; doi:10.1186/s13075-020-02365-y)
Supplement: Supplementary file 4 — Additional file 4: Supplementary Table S4. The results of survival analysis by using Kaplan-Meier analysis in 202 patients with Takayasu’s arteritis in inactive group (Fig. 3A-D) at baseline with further follow-up data. [file 13075_2020_2365_MOESM4_ESM.docx]

**Supplementary table – S4 The results of survival analysis by using Kaplan-Meier analysis in** **202 patients with Takayasu’s arteritis in inactive group (Figure 3A-D) at baseline with further follow-up data.**

| **Patients numbers (N=202)** | | | | **Median duration to relapse (weeks)** | **95%Confidence interval (weeks)** | **P-value** |
| --- | --- | --- | --- | --- | --- | --- |
| 3A | ESR | (≤20 mm/1^st^hr) | 111 | | 97-117 | <0.001 |
|  |  | (>20 mm/1^st^hr) | 59 | | 41-81 |  |
| 3B | hsCRP | (≤8 mg/L) | 113 | | 78-130 | <0.001 |
|  |  | (>8 mg/L) | 79 | | 39-90 |  |
| 3C | IL-6 | (≤5.9 pg/ml) | 117 | | 103-* | <0.001 |
|  |  | (>5.9 pg/ml) | 64 | | 42-91 |  |
| 3D | TNFα | (≤8.1 pg/ml) | 114 | | 109-137 | <0.001 |
|  |  | (>8.1 pg/ml) | 65 | | 44-90 |  |

* The estimation was restricted because of limited sample size of 131 and censored 80 of them.
